# A prescription of information – promoting symptom self-management in people with functional neurological disorder (FND)

**DOI:** 10.1192/bjo.2021.493

**Published:** 2021-06-18

**Authors:** Hashim Dadah, Uzoma Anthony-Uzoeto, Sadat Yazdouni, Ali Aweis-Asanga, Alan Dunlop, Rafey Faruqui

**Affiliations:** 1 King's College London; 2 Kent and Medway NHS and Social Care Partnership Trust; 3 Kent and Medway NHS and Social Care Partnership Trust, University of Kent

## Abstract

**Aims:**

Functional Neurological Disorder (FND) is known to be associated with high healthcare resource utilisation and poor quality of life. Patients’ understanding of the disorder is considered instrumental in improving prognosis.

We produced a symptom self-management patient education strategy with a booklet and FND symptoms recording template in a community neuropsychiatry setting. We embedded this psychoeducation intervention in a post-nursing triage model of care.

**Method:**

A co-production cycle of patient education material was implemented as part of a Quality Improvement Project (QIP) at East Kent Neuropsychiatry Service. Year 4 medical students completed their first QIP cycle involving 4 students, 2 multidisciplinary team members and 4 patients with functional neurological presentations. An FND leaflet and symptom recording template was produced and reviewed using feedback domains such as leaflet readability, perceived usefulness, and template design. The revised version of leaflet was then pilot-tested in second QIP cycle via email or post to 12 patients awaiting their group psychology or neuropsychiatry appointments for treatment of FND. The uptake and impact of leaflet was assessed using telephone-based structured feedback collection.

**Result:**

The first QIP cycle included 10 participants and generated qualitative knowledge domains, providing examples of different types of FND presentations and a biological-psychological-social model explaining onset and/or recurrence of FND symptoms. Group patient feedback and co-production input allowed inclusion of the patient voice and a re-design of leaflet and symptom recording template.

The second QIP cycle involved 12 participants: feedback was collected two weeks after circulation of patient education material. Only 5 participants (42%) had read and used their education leaflet and template during this period. Patients described the booklet as useful overall, but thought it to be more useful at the point of diagnosis and referral to neuropsychiatry. Qualitatively, patients wished there to be more emphasis on FND being explained as “less psychiatric, more a neuropsychiatric problem”, and that it would be “very good for someone who had just been diagnosed”. 80% of responders rated the leaflet quality 8/10 or above. These respondents felt that the leaflet had helped them understand their condition better than they did previously. Usefulness of an additional self-formulation flowchart was rated as 8/10 or below by all patients - with several finding it difficult to use.

**Conclusion:**

Our QIP supports the need for early patient education when discussing diagnosis of FND. The finding of 42% uptake within two weeks of leaflet dispatch is encouraging.

